# Total Knee Arthroplasty and the Evolution of Coronal Alignment: From Mechanical to Personalized Strategies

**DOI:** 10.3390/jpm15110524

**Published:** 2025-11-01

**Authors:** Virginia Cinelli, Marina Marescalchi, Aurelio Picchi, Gerardo De Mattia, Luca Andriollo, Andrea Fidanza, Giandomenico Logroscino, Rudy Sangaletti, Francesco Benazzo, Stefano Marco Paolo Rossi

**Affiliations:** 1 Unità di Chirurgia Robotica, Ortopedia e Traumatologia, Fondazione Poliambulanza Istituto Ospedaliero, 25124 Brescia, Italy; virginia.cinelli02@icatt.it (V.C.); maresc22@gmail.com (M.M.); gerardo.dema@gmail.com (G.D.M.); rudy.sangaletti@poliambulanza.it (R.S.); francesco.benazzo@poliambulanza.it (F.B.); stefano.rossi@poliambulanza.it (S.M.P.R.); 2 Dipartimento di Scienze Geriatriche e Ortopediche, Università Cattolica del Sacro Cuore, 00168 Rome, Italy; 3 Unit of Orthopedics, Department of Life, Health and Environmental Sciences, University of L’Aquila, 67100 L’Aquila, Italy; andrea.fidanza@univaq.it (A.F.); giandomenico.logroscino@univaq.it (G.L.); 4 Ortopedia e Traumatologia, Università Degli Studi di Parma, 43125 Parma, Italy; 5 Artificial Intelligence Center, Alma Mater Europaea University, 1090 Vienna, Austria; 6 Biomedical Sciences Area, IUSS University School for Advanced Studies, 27100 Pavia, Italy; 7 Department of Life Science, Health, and Health Professions, Link Campus University, 00165 Rome, Italy

**Keywords:** total knee arthroplasty, alignment, mechanical, functional, kinematic, positioning

## Abstract

Total knee arthroplasty (TKA) remains a cornerstone of orthopedic surgery, with optimal coronal alignment playing a pivotal role in determining both clinical outcomes and implant longevity. Traditionally, mechanical alignment has been regarded as the gold standard. However, the emergence of alternative philosophies—such as kinematic alignment and hybrid techniques—has shifted the focus toward individualized approaches. Recent advancements in robotic and computer-assisted systems have significantly enhanced the precision of implant positioning, allowing surgeons to better replicate native knee biomechanics and improve patient satisfaction. This narrative review examines current alignment philosophies in TKA, including mechanical, kinematic, and hybrid methods. It analyzes each technique’s principles, functionalities, benefits, and limitations while highlighting ongoing debates regarding their clinical application. Special attention is given to the role of technology in enabling more accurate, patient-specific surgical execution. Despite promising developments, challenges remain in standardizing these techniques and validating their long-term efficacy. To ensure a comprehensive evaluation relevant literature was reviewed, focusing on studies that explore alignment strategies, biomechanical outcomes, and the integration of technology in TKA. This review aims to synthesize current evidence, identify gaps in knowledge, and outline directions for future research needed to optimize alignment strategies in modern knee arthroplasty.

## 1. Introduction

**Figure 1:**
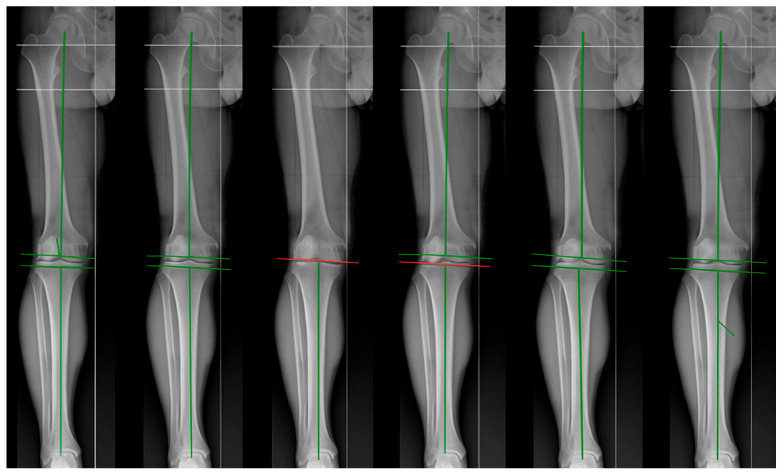
From left to right: anatomic alignment (AA), kinematic alignment (KA), inverse kinematic alignment (iKA), restricted kinematic alignment (rKA), adjusted mechanical alignment (aMA), and functional alignment (FA).

Knee osteoarthritis is a common medical condition in adults, especially among older adults [1,2]. Its incidence and prevalence are rising globally [2,3]. Total knee arthroplasty (TKA) is considered one of the most effective treatments for advanced osteoarthritis generally providing favorable outcomes and enabling patients to resume daily activities. However, up to 10% of patients may still experience unsatisfactory outcomes despite advances in surgical techniques and rehabilitation [1]. The success of TKA is measured by achieving a pain-free knee, good functional outcomes, patient satisfaction, and the long-lasting durability of the implant [3]. Optimal knee joint alignment is regarded as a crucial factor for the durability of the implant. Proper alignment reduces mechanical stress on both the prosthesis and the bone–implant interface. Systematic alignment strategies include mechanical alignment (MA) and anatomic alignment (AA), which aim to restore a neutral hip-knee-ankle (HKA) axis of 180° for all patients regardless of pre-operative alignment [3]. In contrast, patient-specific alignment, such as kinematic alignment (KA), focuses on maintaining the native limb alignment and joint line inclination [3]. Lastly, hybrid alignment—encompassing restricted kinematic alignment (rKA), inverse kinematic alignment (iKA), adjusted mechanical alignment (aMA), and functional alignment (FA)—aims to restore coronal alignment within a safe HKA angle range of 177° to 183° (Figure 1) [4]. This review provides a comprehensive overview of the evolution of coronal alignment strategies in total knee arthroplasty, comparing traditional approaches with newer patient-specific and hybrid techniques, and discussing their clinical implications and supporting evidence.

## 2. Mechanical Alignment

The mechanical axis of the lower limb is an imaginary line that extends from the center of rotation of the femoral head to the center of the ankle. It can be divided into two parts: the femoral mechanical axis and the tibial mechanical axis. The femoral mechanical axis is drawn from the center of rotation of the femoral head to the center of the knee joint. In contrast, the tibial mechanical axis is drawn from the knee joint’s center to the ankle’s center. When these two lines are extended, they converge at the center of the knee, forming the HKA angle. Typically, this angle measures a few degrees less than 180° [1]. MA approach in TKA, introduced by Insall and Ranawat in the 1970s, involves making a femoral cut and a tibial cut perpendicular to the mechanical axes of the femur and tibia, respectively [5,6]. They believed that anatomical alignment could lead to failure of fixation of the medial tibial plateau due to uneven distribution of forces [6]. They also recommended externally rotating the femoral component by 3° in TKA to balance the flexion and extension gaps [5]. The femoral component is typically aligned with the trans-epicondylar axis, which serves as the reference axis for flexion and extension movements in the knee [5]. The optimal distal femoral resection is usually performed at a valgus angle of 2° to 7° relative to the femoral shaft axis [5]. This alignment in valgus helps achieve a neutral MA of the knee joint [7]. Restoring a neutral mechanical axis is commonly believed to improve implant longevity and patient function following TKA [8]. By ensuring load symmetry, this alignment prevents uneven wear of the polyethylene insert, which had been a significant issue and frequent complication in the past [9,10]. Studies have demonstrated that both computer-assisted MA-TKA and robot-assisted MA-TKA result in better mechanical axis alignment and more accurate component placement than traditional surgery [7,8,9,10,11]. For example, the robotic system achieved MA with reliable radiographic outcomes and clinical results in the treatment of major deformities of the lower limb with the use of more constrained implants at short-term follow-up [12]. However, despite these improvements, functional outcomes remain unsatisfactory for some patients, with approximately 10% continuing to experience symptoms such as pain, instability, stiffness, and swelling after MA-TKA [12]. While this alignment range is considered optimal, it is not the only factor influencing functional success [11]. The primary goal of MA is to achieve a neutral HKA axis, where the mechanical axes of the femur and tibia ideally form an angle of 180°, signifying perfect knee alignment [11]. However, it has been observed that only a small percentage of individuals are naturally aligned in this way. MacDessi et al. found that only 15.4% of normal knees and 14.6% of arthritic knees have a mechanically neutral alignment and joint line [13]. Bellemans et al. demonstrated that over 30% of male patients without arthritis had a constitutional varus angle greater than 3° [14]. Similarly, Hirschmann et al. found a considerable variation in femoral and tibial coronal alignment in young knees that were not affected by osteoarthritis [15]. Therefore, aligning the tibial and femoral components perpendicular to the mechanical axis may result in an alignment that differs from the natural knee, causing abnormal joint line inclination and altering the normal biomechanics of the knee, which could have adverse effects on clinical outcomes in TKA. This indicates that for most patients (around 85%), mechanically neutral joint replacement does not fully restore the native alignment of the knee, and there is a need for further exploration of alternative alignment methods and strategies to improve outcomes for these patients [16].

## 3. Anatomical Alignment

The anatomical axis of the femur is represented by an imaginary line that extends from the proximal end to the distal end of the femur, passing through the diaphysis at a point equidistant from the medial and lateral cortices [17]. The mechanical axis and the anatomical axis converge toward the center of the knee, forming an angle known as the Anatomical Mechanical Angle (AMA), which typically measures between 5° and 6°. The anatomical axis of the tibia aligns with its mechanical axis. The HKA angle, formed by the intersection of the two anatomical axes at the knee, is essential for assessing various deformities. The normal range for this angle is between 173° and 175°. The first concept of anatomical alignment (AA) in TKA was introduced by Hungerford and Krackow in 1985 [17]. They suggested that the knee prosthesis components should be positioned to closely resemble the natural anatomy of the knee joint and the oblique joint line [17]. AA aims to position the femoral component at a fixed angle of 3° valgus and the tibial component at 3° varus relative to the limb’s mechanical axis. This approach allows restoration of the native knee anatomy and the natural joint line inclination of 3° while maintaining periarticular soft-tissue tension throughout the full range of motion [17]. By properly releasing the ligaments, overall limb alignment is restored to neutral (HKA 180°) for all patients, which qualifies it as a systematic approach [4]. The approach aims to restore the limb’s overall alignment to a neutral position, eliminating the need for external rotation of the femoral component [17]. The rationale supporting this technique is improved load distribution across the tibial component and better biomechanics of the patellofemoral joint [18]. While the short-term results of this technique have been generally positive, it is important to note that catastrophic wear of the polyethylene component has been reported in about 5% of cases at long-term follow-up, leading to the need for revision surgery [7,18]. However, a study by Yeo et al. showed that the wear characteristics and durability of modern polyethylene components have significantly improved [19]. The use of robotics in TKA also allows for more accurate alignment of implant components, which contributes to better functional outcomes and enhanced joint stability [19]. Previously, achieving precise osteotomies posed technical challenges, such as the risk of excessive varus alignment or incorrect positioning of the tibial implant [20].

## 4. Adjusted Mechanical Alignment

The adjusted mechanical alignment (aMA) technique addresses slight constitutional knee deformities, preserving up to 3° of deformity [21]. As an extension of conventional MA, aMA aims to create a wider lateral flexion–extension gap and reduce soft tissue releases, making it suitable for patients with constitutional knee deformity [21]. Implant adjustments are made on the femoral side, while the tibial component is aligned at 90° to the mechanical axis. The aMA strategy allows for the preservation of frontal plane deformities within a range of up to 6° of varus or valgus, thus minimizing the need for ligament releases [21]. Graichen et al. demonstrated that aMA in TKA effectively achieved target HKA angles and overall gap balance, although equal flexion–extension gap sizes were less consistently obtained [22]. Significant non-anatomical bone resections were often required, especially in the medial tibial plateau and lateral posterior condyle, particularly in varus knees and valgus knees [3,23,24]. Approximately 50% of all knee types required non-anatomical resections of the lateral posterior condyle, which has been associated with worse clinical outcomes [23,24]. The authors linked these non-anatomical modifications to potential patient dissatisfaction, advocating for more individualized and anatomically respecting surgical approaches [23,24].

## 5. Kinematic Alignment

Knee kinematics involve the relative motion and positioning of the femur, patella, and tibia as the flexion angle changes, without considering the forces acting on the knee [24]. Kinematic alignment (KA) is defined by three functional axes that describe movements of knee joint [25]. The first kinematic axis is the femoral transverse axis found in the femur, serving as the pivot around which the tibia performs flexion and extension. This axis aligns with the center of a circle that matches the articular surface of the femoral condyles [25]. The second kinematic axis refers to the transverse axis of the tibia, which serves as the pivot for the patella’s flexion and extension on the femur. This axis is located proximally and anteriorly, and it runs parallel to the primary transverse axis [25]. The third axis is the longitudinal axis of the tibia, which allows the tibia to rotate internally and externally relative to the femur. This axis is perpendicular to the first two axes. Together, these three kinematic axes define the complex and dynamic movements of the knee, including flexion, extension, patellar movement, and tibial rotation [24]. Defining standardized safe zones in KA remains challenging, particularly in obese patients, as increased soft tissue and altered anatomy can complicate component positioning [25]. These factors emphasize the need for careful preoperative planning and intraoperative assessment to ensure safe and effective alignment in complex or high-BMI knees condyles [25]. The KA technique, introduced by Howell et al. in 2008, is a “true femoral resurfacing method” [25]. In this three-dimensional approach, TKA implants are placed anatomically to closely match the natural knee of patient [26]. Similarly to partial knee replacements, KA is considered a patient-specific and ligament-sparing approach, focusing on bone preservation [24]. Its goal is to restore the unique, pre-arthritic alignment of the limb and joint line, which can vary significantly between individuals [18]. Furthermore, KA aims to address knee laxity without requiring complex preoperative evaluations or planning [7]. By restoring the knee joint’s natural alignment and movement, KA may improve range of motion, joint stability, and overall function [26]. Additionally, KA helps achieve normal patellar tracking by preserving the native Q angle, which is the angle formed between the quadriceps tendon and the patellar tendon [25,27,28,29]. Moreover, KA has been associated with less postoperative pain compared to other alignment methods [30]. This approach is particularly beneficial for knees with valgus deformity, as it reduces the need for extensive soft tissue manipulation and simplifies the surgical procedure [7]. A 2012 study by Dossett et al. found that after six months, the KA group showed significant improvements compared to the MA group [26]. The KA group had a 16-point increase in the WOMAC score, a 7-point increase in the Oxford Knee score, and a 25-point increase in the combined Knee Society score, along with 5° more flexion [26]. These results indicate that KA leads to better functional outcomes. However, a meta-analysis by Courtney and Lee found similar complication rates between KA and MA-TKA after two years [31]. Most of the revision surgeries in that study (1.2% of all cases) were related to patellofemoral issues. A ten-year follow-up study showed KA’s long-term durability, with a survival rate of 97.5%, underscoring the technique’s effectiveness in TKA [32]. In another study by Shelton et al., satisfaction rates were assessed in patients who had one knee treated with a kinematically aligned TKA and the other with a mechanically aligned TKA [33]. Before switching to the KA approach, 83% of patients expressed satisfaction with the mechanically aligned TKA [34]. After transitioning to KA, patient satisfaction increased to 92% during the follow-up period [35]. Similarly, Bellemans et al. explored the use of KA in TKA tailored to the individual knee’s native anatomy and found that KA significantly improved early clinical outcomes, including pain relief and functional recovery, surpassing traditional alignment methods [14]. This highlights the personalized benefits of KA in TKA [14,34]. Although KA has shown promising results, the optimal range for component positioning remains uncertain [14,34]. It is crucial to define an effective and safe range for positioning the components in KA. Moreover, the biomechanics of an osteoarthritic knee may differ from those of a pre-arthritic knee, which adds complexity to the alignment process in KA [7]. While MA has been extensively studied with long-term follow-up data, KA has not been studied as comprehensively. The relatively short follow-up periods in KA studies limit our understanding of its long-term outcomes, and further research with extended follow-up could provide valuable information on the technique’s potential impact on long-term results [34].

## 6. Restricted Kinematic Alignment

In 2011, Vendittoli introduced an innovative approach known as rKA protocol, addressing the challenges associated with complex anatomies and significant knee alterations [35]. This protocol aims to perform bone resections according to the principles of KA in most cases but also provides the flexibility to make adjustments for patients who fall outside a “safe range”. The guidelines for this range establish that femoral and tibial cuts must remain within 5° of the mechanical axes of the respective bone and that the resulting HKA angle should not deviate by more than 3° from neutral [35]. This approach is particularly beneficial in cases of severe arthritic changes, trauma, tumor-related conditions, previous surgeries, and congenital deformities [36]. The primary goal of rKA is to preserve the native anatomical structure of the knee while maintaining ligamentous balance and minimizing the risk of postoperative instability [35]. Additionally, it enables surgeons to perform interventions in a more conservative and precise manner, optimizing clinical outcomes for patients undergoing TKA [35]. In summary, the rKA approach represents an effective compromise, ensuring a safe alignment of the lower limbs while promoting the long-term well-being of patients [35]. However, there are still few studies on the medium- to long-term clinical outcomes [36]. Abhari et al. found that rKA with advanced technology in primary TKA resulted in significantly higher patient satisfaction (93% vs. 81%, *p* < 0.001) and better functional outcomes (KOOS, WOMAC, FJS, Knee Society scores) at 17 months compared to conventional neutral MA [37]. The authors concluded that rKA with advanced technology offers excellent results [37]. Shichman et al. demonstrated that the rKA-TKA showed superior accuracy in restoring both the joint line obliquity (JLO)—the angle formed between the proximal tibial joint line and the floor—and joint line height (JLH), which is measured as the distance from the femoral articular surface to the adductor tubercle [38]. The study found that rKA-TKA exhibited less variation in JLO pre- and post-operatively compared to MA-TKA. Given the increasing emphasis on precise joint line restoration, these findings underscore the potential advantages of rKA-TKA over MA-TKA [38].

## 7. Inverse Kinematic Alignment

Winnock de Grave introduced iKA in 2022, a tibia-first, gap balancing technique in which the proximal tibial resection is performed first, followed by the femoral resection. This approach can be implemented as either pure kinematic alignment or rKA and aims to restore the native tibial joint line obliquity [39]. The technique, demonstrated using robotic surgery to enhance resection precision, is particularly beneficial in cases of severe wear on the medial tibial plateau [39]. In KA, the initial femoral cut can sometimes lead to excessive tibial resections and varus alignment as a compensatory mechanism, increasing risk of implant failure [39]. In contrast, iKA aims to restore the pre-arthritic medial proximal tibial angle (MPTA) within the safe zone of 84° (varus) to 92° (valgus), while also maintaining the original slope [39]. This produces a tibial joint line with a postoperative varus alignment of just 3° ± 2°, which is more favorable because it uses a tibial reference rather than a femoral one [4]. Genesatux et al. demonstrated that in a study of 100 robotic TKAs utilizing inverse kinematic alignment, excellent one-year clinical patellofemoral outcomes were observed (mean Kujala score 85.69, HSS Patellar score 88.15), comparable to preoperative scores [40]. Although clinically significant improvement was noted, no substantial radiological changes in patellar tilt or lateralization were detected. The combined technique shows promise for improving patellofemoral function; however, further research is needed to confirm the radiological benefits [40]. Even with conventional instrumentation, the iKA technique provides good clinical and radiological outcomes in restoring the constitutional alignment of the lower limb [41]. Analysis of 972 healthy knees showed iKA accommodates a significantly larger proportion (74%) than aMA (8%) or rKA (55%) in TKA. iKA also requires fewer adjustments to achieve optimal implant placement, suggesting its potential for improved surgical outcomes [42].

## 8. Functional Alignment

FA is an evolution of kinematic alignment that focuses on restoring ligament tension throughout the range of motion. Its precise implementation relies on advanced technologies, such as robotic or navigation systems, to accurately achieve the desired joint alignment [43]. KA focuses on replicating the pre-existing articular surface, while FA emphasizes joint space balancing throughout the knee’s range of motion [44]. Understanding personalized alignment in the knee is facilitated by a three-element model encompassing morphology, alignment, and soft tissue optimal function, arising from the harmonious interplay of these elements, where each influences and is influenced by the others, ultimately determining individual knee kinematics [43]. Individualized alignment in TKA uses real-time intraoperative assessment of resection thickness, joint spaces, and limb alignment to optimize implant positioning. Robotic technology enhances precision, ensuring reproducible and accurate alignment while harmoniously balancing the knee in three dimensions [43]. The approach integrates controlled resection and gap-balancing techniques. Alignment is assessed before and after osteophyte removal, with coronal correction evaluated at various flexion angles under varus/valgus stress to optimize ligamentous balance. Software calculates joint space dimensions (in extension and at 90° flexion) and assesses ligamentous laxity, providing immediate feedback to the surgeon on the effects of bone cuts and ligament releases [43]. Gap balancing is achieved by adjusting implant targets. The surgeon selects the optimal solution for each patient, compensating for any imbalances through bone cut adjustments while minimizing ligament releases. Robotic assistance ensures safe and controlled alignment, within parameters that may be further refined by future research. Varus or valgus corrections can be applied to the femur and tibia to achieve the desired joint line obliquity [43]. Joint line height is maintained, reducing instability risks. The overall goal is optimal prosthetic positioning. Even in complex deformities, ligament releases are minimized compared to traditional MA techniques, thanks to continuous real-time feedback [45]. Preliminary findings indicate both the precision of these systems and favorable clinical and radiological outcomes resulting from their application [46]. An individualized alignment plan can achieve a final implant position with increased joint line obliquity while maintaining overall limb alignment. This method has shown promising results, particularly in patients with constitutional varus, leading to better outcomes two years after surgery [47]. Jeffrey et al., in a retrospective study, compared FA and aMA in 128 TKAs using a robotic system. Patients in the FA group demonstrated significantly better short-term clinical outcomes (FJS, OKS, KSS scores) at final follow-up compared to the aMA group, and FA patients also reported fewer instances of instability [48]. While the difference in postoperative HKA angle between FA and aMA groups was statistically significant, the authors suggest the clinical significance is uncertain due to the small magnitude of the difference (177.3° ± 2.0 vs. 178.2° ± 2.0) [48]. Parratte et al. compared anatomic-functional implant positioning with aMA in 40 robotic TKA procedures, which aims for physiological ligament balance (symmetric extension gap, asymmetric flexion gap), demonstrated comparable postoperative HKA angles to aMA [48]. This resulted in a more natural ligamentous pattern, suggesting equivalent long-term clinical outcomes with faster recovery compared to aMA [48]. It is crucial to conduct long-term follow-ups to assess both clinical outcomes and the longevity of the implants associated with these modern techniques. However, potential drawbacks such as the learning curve, increased procedural cost, and limited accessibility in low-resource settings should also be considered when evaluating the adoption of robotic FA [48,49].

## 9. Discussion

**Figure 2:**
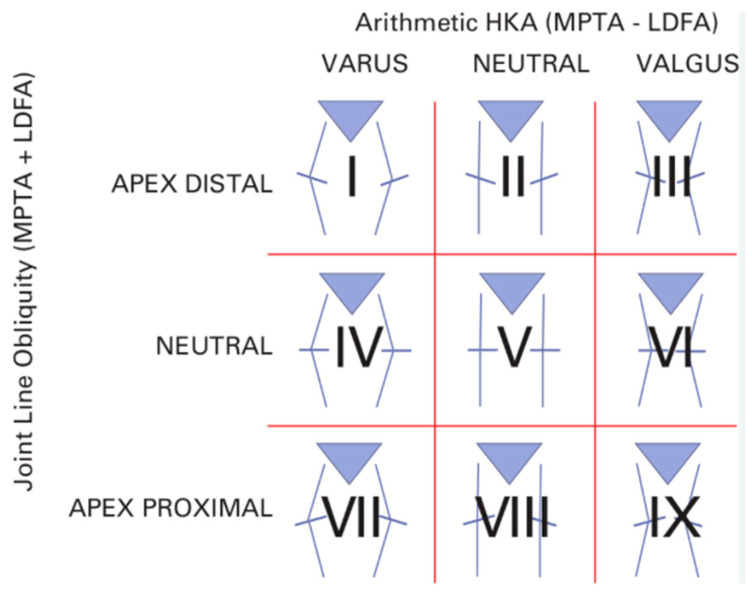
The C-PAK classification identifies nine distinct cellular phenotypes based on morphological and functional characteristics [13].

In TKA, achieving optimal coronal alignment is crucial for enhancing both short-term and long-term outcomes. The Coronal Plane Alignment of the Knee (CPAK) classification is a practical and intuitive way to describe how a knee is naturally aligned (Figure 2); this system considers two main aspects of alignment: HKA, which can be varus, neutral, or valgus, and the JLO, which refers to the angle of the knee’s joint surface—whether it tilts downward (distal apex), remains level (neutral), or tilts upward (proximal apex) [13]. The most represented subtypes are II, I, and IV, both in healthy individuals and in those with osteoarthritis [50].

Subtype II (neutral HKA and distal apex JLO) is the most common variant, representing about 40% of the population, and represents a practical example of the Hungerford principle related to AA [17]. However, even though this method aimed to properly align the JL, achieving this goal with conventional instruments proved difficult, which led to its gradual abandonment [17]. It has also been shown that, particularly for subtypes I, II, and III, additional bone recuts are more often required with MA compared to KA [51]. Furthermore, Corban et al. demonstrated that under a fixed MA approach, coronal alignment remains unchanged, but the joint line angle increases significantly [52]. Among 700 knees, 60% (420 knees) were realigned from their constitutional phenotype to CPAK Type V (the MA target), 35% (245 knees) shifted to a phenotype different from Type V, and only 5% (35 knees) retained their original CPAK type [52]. In a 2019 study of 210 consecutive TKAs, KA achieved the same sagittal correction as MA but required less bone removal and fewer soft tissue releases [53]. Clark et al. showed that the MA approach increased the tilt of the joint line without significantly changing coronal alignment. They also found that altering the JLO in CPAK Type I knees worsened the clinical outcomes of TKA [47]. Conversely, Agarwal et al. showed that when using robot-assisted MA-TKA, modifications to the patient’s native joint line and CPAK classification do not have a significant effect on patient satisfaction one year after the surgery [54]. Therefore, while MA was considered the standard for many years, today the suboptimal results of this technique have been highlighted. Newer techniques, including KA, hybrid approaches (rKA, iKA, aMA), and FA, offer more personalized options aimed at restoring native knee biomechanics. This, in turn, can enhance patient satisfaction, reduce postoperative complications, and improve implant longevity. Hirschmann et al. showed significant variability in knee alignment in a 3D CT scan analysis of non-osteoarthritic knees. Females displayed more valgus alignment than males [55]. Neutral femoral alignment was common in both sexes, but neutral tibial alignment was more frequent in males, while valgus tibial alignment was more frequent in females [55]. The same research group found substantial variability in osteoarthritis patient lower limb alignment, with more varus alignment in males. Current TKA alignment techniques often neglect the significant variations in HKA, FMA, TMA, and JLCA, emphasizing overall limb alignment instead of individual joint line characteristics, may contribute to patient dissatisfaction [56]. This highlights the need for individualized TKA approaches. While robotic and computer-assisted surgeries have increased the precision of alignment and component placement, further studies are necessary to evaluate the long-term impact of these techniques on implant survival and functional recovery. The shift toward individualized treatment, which addresses each patient’s unique anatomical and functional requirements, appears promising. Finally, the current discourse surrounding TKA alignment predominantly centers on coronal plane alignment. However, emerging evidence suggests that sagittal plane alignment, encompassing parameters such as the flexion angle of the femoral component and the posterior slope of the tibial component, may exert a significant influence on functional outcomes. Nedopil et al. demonstrated that patellofemoral instability following kinematically aligned TKA is infrequent, occurs without trauma, and is correlated with an increased flexion of the femoral component [57]. Adequate restoration of the third space in TKA requires matching trochlear component depth to native trochlear groove height. However, significant variation exists in trochlear groove height relative to the anterior femoral cortex. With a standard 2.2 mm trochlear depth, over- or under-stuffing exceeding 2 mm is expected in approximately 24.5% of patients. This variability is further compounded by variations in femoral component flexion and extension, and whether the component is left proud or notched.

Failure to accurately restore the third space is likely to contribute to poor TKA outcomes [58]. The effect of KA and other PA techniques on the patellofemoral joint remains a subject of ongoing debate, emphasizing the need for further investigation [23]. To fully realize the potential benefits of these newer, more patient-specific alignment methods, the development and utilization of prosthetic implants specifically engineered for PA are crucial. The concept of alignment on the coronal plane is evolving, with Koutserimpas et al. and Andriollo et al. introducing the concept of “functional positioning”, a 3D approach in which the sagittal and axial planes also play a significant role, along with the introduction of the HKA concept at 90° of flexion [59,60,61,62,63,64]. Table 1 summarizes the main alignment strategies in total knee arthroplasty, highlighting their principles, advantages, and limitations to provide a concise comparison of the different approaches. Although KA, FA, and hybrid approaches show promising early results, long-term outcome data remain limited, making it difficult to fully assess their durability and effectiveness compared to traditional MA [65,66,67]. Furthermore, controversies persist regarding the optimal indications for each technique, and potential risks such as altered JLO or over-reliance on patient-specific alignment, require further investigation.

## 10. Conclusions

In conclusion, the choice of alignment method should be tailored to the individual patient’s needs, considering their preoperative deformity and desired functional outcomes. As technology continues to advance, there is a significant chance that TKA will become even more precise, patient-centered, and effective, leading to better overall postoperative results. Recently introduced alignment techniques require time to demonstrate their anticipated benefits, while long-term data for MA and AA confirm reliable outcomes but do not resolve issues such as the persistent approximately 10% patient dissatisfaction after TKA.

## Figures and Tables

**Table 1:** Overview of main alignment strategies in TKA, summarizing their principles, advantages, and limitations.

Approach	Principle	Advantages	Limitations
Mechanical Alignment (MA)	Restores neutral HKA = 180°, cuts perpendicular to mechanical axes.	Standardized, strong long-term survival data, implant longevity.	Does not respect native anatomy in most patients, ~10% dissatisfaction.
Anatomical Alignment (AA)	Reproduces the anatomical axis with physiological joint line obliquity.	Respects native anatomy, improves patellofemoral biomechanics.	Historically associated with polyethylene wear, requires high surgical accuracy.
Kinematic Alignment (KA)	Recreates pre-arthritic patient-specific alignment (“true resurfacing”).	Ligament-sparing, bone-preserving, improved ROM, satisfaction, and pain relief.	Safe zones not standardized, limited long-term data.
Adjusted Mechanical Alignment (aMA)	Maintains up to 3° of constitutional deformity, reducing releases.	More personalized than MA, reduces non-physiological resections.	Still requires non-anatomical resections in many cases, potential dissatisfaction.
Restricted Kinematic Alignment (rKA)	KA within defined safety boundaries (±5° from mechanical axes).	Compromise between personalization and safety, higher satisfaction rates.	Mid- to long-term outcomes still under investigation.
Inverse Kinematic Alignment (iKA)	“Tibia-first” technique restoring tibial joint line obliquity.	Precise with robotic assistance, better patellofemoral outcomes.	Novel approach, limited long-term validation.
Functional Alignment (FA)	Focus on real-time ligament balancing with robotic/navigation guidance.	3D individualized alignment, fewer releases, favorable early outcomes.	Requires advanced technology, higher costs, learning curve.

## Data Availability

No new data were created or analyzed in this study. Data sharing is not applicable to this article.
